# Extreme Geochemical Conditions and Dispersal Limitation Retard Primary Succession of Microbial Communities in Gold Tailings

**DOI:** 10.3389/fmicb.2018.02785

**Published:** 2018-11-28

**Authors:** Talitha C. Santini, Maija Raudsepp, Jessica Hamilton, Jasmine Nunn

**Affiliations:** ^1^School of Agriculture and Environment, The University of Western Australia, Crawley, WA, Australia; ^2^School of Earth and Environmental Sciences, The University of Queensland, St Lucia, QLD, Australia; ^3^Department of Earth and Atmospheric Sciences, University of Alberta, Edmonton, AB, Canada

**Keywords:** microbial community succession, tailings, rehabilitation, microbial community assembly, dispersal, microbial colonization

## Abstract

Microbial community succession in tailings materials is poorly understood at present, and likely to be substantially different from similar processes in natural primary successional environments due to the unusual geochemical properties of tailings and the isolated design of tailings storage facilities. This is the first study to evaluate processes of primary succession in microbial communities colonizing unamended tailings, and compare the relative importance of stochastic (predominantly dust-borne dispersal) and deterministic (strong selection pressures from extreme geochemical properties) processes in governing community assembly rates and trajectories to those observed in natural environments. Dispersal-based recruitment required > 6 months to shift microbial community composition in unamended, field-weathered gold tailings; and in the absence of targeted inoculants, recruitment was dominated by salt- and alkali-tolerant species. In addition, cell numbers were less than 10^6^ cells/g tailings until > 6 months after deposition. Laboratory experiments simulating microbial cell addition *via* dust revealed that high (>6 months’ equivalent) dust addition rates were required to effect stabilization of microbial cell counts in tailings. In field-weathered tailings, topsoil addition during rehabilitation works exerted a double effect, acting as a microbial inoculant and correcting geochemical properties of tailings. However, microbial communities in rehabilitated tailings remained compositionally distinct from those of reference soils in surrounding environments. pH, water extractable Mg, and water extractable Fe emerged as major controls on microbial community composition in the field-weathered gold tailings. Overall, this study highlights the need for application of targeted microbial inoculants to accelerate rates of microbial community succession in tailings, which are limited primarily by slow dispersal due to physical and spatial isolation of tailings facilities from inoculant sources; and for geochemical properties of tailings to be amended to moderate values to encourage microbial community diversification and succession.

## Introduction

Approximately 7 Gt of tailings are produced globally each year as wastes and by products of mineral extraction and beneficiation processes, with gold tailings accounting for one-fifth (1.27 Gt) of this total (Mudd and Boger, 2013). Gold tailings are typically produced at an alkaline pH with high salinity and sodicity (average EC: 4 mS cm^-1^, average exchangeable sodium percentage: 57%), low to undetectable concentrations of total organic carbon (<0.4% wt) and other nutrients (Bell et al., 1999; Zagury et al., 2004; Mains et al., 2006; Lindsay et al., 2009; Truong et al., 2010; Ni et al., 2014; Santini and Banning, 2016), and are fine grained with a high moisture content tending toward waterlogging (Ni et al., 2014; Santini and Banning, 2016). Where gold is hosted in sulfide-bearing deposits, pyrite, and other sulfides in the tailings can oxidize, resulting in acid generation and pH decrease after deposition in tailings storage facilities (Ritcey, 2005; Lindsay et al., 2009). Remediating the extreme geochemical properties of tailings is the focus of rehabilitation strategies designed to convert tailings into a soil-like medium, and ultimately to create a material that can support a stable, self-sustaining ecosystem after mine site closure. In the absence of targeted rehabilitation or amendment strategies, *in situ* natural weathering of the tailings, including combined physical, chemical and microbial processes, can take 50 years or more to decrease and stabilize the pH and decrease salinity to plant-tolerable values (pH 5.5–8; EC < 1 mS cm^-1^; Londry and Sherriff, 2007; Young et al., 2012). As limiting chemical, physical, and biological conditions abate during natural weathering, spontaneous colonization of the tailings by pioneer vegetation species (typically grasses) may occur, followed by shrubs and trees, which in turn supports *in situ* accumulation of organic carbon and nitrogen and development of stable soil aggregates (Londry and Sherriff, 2007; Young et al., 2012).

Most studies to date have focused on the role of vegetation in driving *in situ* rehabilitation and the establishment of a stable, self-sustaining ecosystem in tailings (Young et al., 2012, 2015; Ni et al., 2014, 2015), neglecting the role of microbial communities in *in situ* rehabilitation. Microbial communities can make important contributions to key aspects of tailings rehabilitation including pH neutralization (Santini et al., 2015a, 2016), nutrient accumulation (Banning et al., 2010; Zhan and Sun, 2011; Nelson et al., 2015; Santini et al., 2015a; Li et al., 2016), degradation of tailings-specific contaminants such as cyanide and thiocyanate (Skowronski and Strobel, 1969; Akcil, 2003; Vu et al., 2013), and plant-microbe symbioses (Grandlic et al., 2008; Banning et al., 2010; Solis-Dominguez et al., 2011; Young et al., 2012). Enhancing these beneficial contributions is predicated on an improved understanding of the early stages of microbial community assembly and succession in tailings; in particular, the key geochemical and physical controls on microbial recruitment, growth, and community succession. The process of primary succession in microbial communities in tailings is currently poorly understood, and is likely to be substantially different to that observed in natural primary successional environments, for two main reasons: (a) spatial and physical isolation from inocula; and (b) extreme geochemical properties of tailings. Both the rate of microbial cell influx into the tailings environment, and the tolerances of the incoming inocula for the geochemical and physical properties of the tailings will influence the trajectory and rates of microbial community assembly.

Natural primary successional environments, such as post-wildfire soils and deglaciated till, typically have diffuse boundaries with environments hosting high microbial biomass and diversity communities, and are open to ingress from microbial vectors such as biota and overland flow. These vectors facilitate rapid colonization and microbial community succession, on the order of hours to weeks (Ferrenberg et al., 2013; Nemergut et al., 2016). In contrast, tailings storage facilities are spatially and physically isolated from natural microbial inoculant sources by site infrastructure (refinery plant, roads, firebreaks), and physical containment structures (bunding, drainage channels) for tailings and leachates. This infrastructure prevents overland water flow from carrying suspended soil particulates into the tailings storage facility, and restricts thoroughfare by local biota. With other vectors excluded, dust deposition is therefore likely to be the primary method of microbial cell influx into tailings storage facilities. Relatively little data is available to compare rates of microbial cell influx from different vectors, even in natural primary successional environments; however, potential delivery from dust can be estimated based on local or regional dust deposition rates and observed cell counts in local soils. Incoming dust transported by wind from soil surfaces in areas surrounding the mine and refinery sites will be deposited in tailings storage facilities, inoculating them with native soil microorganisms. Topsoil, from which aeolian dusts would be generated, typically host 10^7^–10^9^ microbial cells per gram (Richter and Markewitz, 1995; Whitman et al., 1998; Griffin, 2007). Globally, dust deposition rates vary between 3 and 1315 mg/m^2^/day (Hingston and Gailitis, 1976; Drees et al., 1993; Ta et al., 2004; O’Hara et al., 2006; Cattle et al., 2009), with Australian dust deposition rates toward the low end of global observations (usually less than 186 mg/m^2^/day; Hingston and Gailitis, 1976; Leys and McTainsh, 1999; Hesse and McTainsh, 2003; Cattle et al., 2009). Studies reporting local or even regional contemporary dust deposition rates near Australian mine sites are scarce, with only one study to date reporting rates near a gold mine site [Cowal Gold, NSW, Australia, 46–164 mg/m^2^/day (Cattle et al., 2012)]. Australian dust emission guideline values match well with national observation data, indicating upper limits of 133 mg/m^2^/day as acceptable dust deposition rates around mine sites and associated activities (Environmental Protection Authority of Victoria, 2007; Department of Industry Innovation and Science [DIIS], 2009; South West System Supply Chain Members [SWSSCM], 2013; New Zealand Ministry for the Environment [NZME], 2016). Compared with natural primary successional environments, low dust deposition rates (despite high potential microbial cell loadings) coupled with removal of other microbial cell vectors through physical and spatial isolation can be expected to substantially decrease microbial colonization rates of tailings storage areas (‘dispersal limitation’) and retard microbial community assembly and succession processes.

Once microorganisms enter the tailings environment, the extreme geochemical properties of tailings compared to bedrock-derived soils also pose challenges for microbial colonization and community succession. Gold tailings differ from other sulfide-bearing tailings in that gold tailings are treated with NaOH as part of the gold extraction process and thus have a high initial pH (usually pH 9–9.5) on deposition in tailings storage areas, which tends toward circumneutral with time as residual sulfides oxidize and release acid (Ni et al., 2014). In contrast, base metal tailings generated from processing of sulfide ores (e.g., chalcopyrite [CuFeS_2_], chalcocite [Cu_2_S], sphalerite [ZnS], galena [PbS]) do not have NaOH added during processing, and usually exhibit an initially circumneutral pH after deposition, which decreases to acidic values (pH ≤ 5.5) over time through the oxidation of residual sulfides and lack of internal acid buffering capacity. Geochemically, gold tailings therefore behave more like other alkaline tailings than other sulfide-bearing tailings, and microbial community successional processes may therefore also be similar to those observed in other alkaline tailings. In tailings undergoing rehabilitation, over 50% of variation in microbial community composition is generally attributed to cumulative geochemical factors, with these factors being cited as the major constraint on microbial community diversity in tailings (Banning et al., 2010; Kuang et al., 2013; Liu et al., 2014; Valentín-Vargas et al., 2014; Santini et al., 2015b)^.^ Most often, pH is identified as the key environmental control, accounting for 14–67% of overall variation in microbial community composition (Banning et al., 2010; Kuang et al., 2013; Liu et al., 2014), with salinity also strongly influencing microbial community composition (Kuang et al., 2013; Santini et al., 2015b). Low concentrations of organic C, N, and P for heterotrophic growth and building microbial biomass are also likely to shape microbial community succession in tailings, although these appear to be secondary constraints to pH and salinity in most studies (Banning et al., 2010; Kuang et al., 2013; Valentín-Vargas et al., 2014). Based on the strength of environmental selection pressures in shaping later stages of succession in tailings microbial communities, microbial inoculants landing in tailings environments *via* dust-borne dispersal are therefore hypothesized to be subsequently filtered by their tolerances to the geochemical properties of tailings.

To date, no studies have investigated primary microbial community succession in tailings in the absence of targeted rehabilitation efforts (addition of amendments). Prior studies have focused on either: identifying geochemical controls on microbial community composition in unamended tailings, typically as a ‘snapshot’ (one time point) study, often without considering the age of the tailings; or, on microbial community succession processes in rehabilitated (amended) tailings. This limits our ability to understand the relative importance of dispersal limitation and selection pressures in controlling rates and direction of microbial community assembly and succession in unamended mine tailings. Consequently, it is difficult to assess the role that microbial communities may play in initial ecosystem establishment in tailings, and justify and optimize the use of targeted amendments to enhance or guide primary microbial community succession. This study therefore aimed to: (a) quantify rates of microbial community succession in unamended tailings; (b) assess whether dust-borne dispersal alone was sufficient to shift microbial community structure in unamended tailings toward that of rehabilitated tailings or natural soils during primary succession; (c) identify major environmental controls (e.g., pH, salinity) on microbial community composition during primary succession; and (d) evaluate the roles of environmental factors (selection) and dispersal limitation in guiding microbial community assembly and primary succession in unamended tailings. Analysis of field samples from an alkaline gold tailings chronosequence in southwest Western Australia, comprising unamended tailings, rehabilitated tailings, and nearby natural soils to examine microbial community composition and tailings geochemistry was supported by laboratory experiments to test survival and growth of microbial inocula arriving *via* dust.

## Materials and Methods

### Field Sampling Location and Sites

Tailings and soil samples were collected from the Newmont Boddington gold mine, near Boddington, WA, Australia. Mining occurs on an Archaean hydrothermal deposit that contains gold, copper, and appreciable amounts of molybdenum hosted in andesite/diorite intrusive and volcanic rocks (McCuaig et al., 2001). Mining and processing of both the weathered laterite sequence and the host bedrock has occurred in the mine’s history. Currently much of the tailings is composed of a kaolinitic clay (Ni et al., 2014). Climate has previously been detailed elsewhere (Ni et al., 2014); this location has a Mediterranean climate with hot dry summers and cool wet winters. It receives an annual average rainfall of 510 mm (Bureau of Meteorology, 2018a), most of which falls in winter (April to October), and has a mean monthly maximum of 32.1°C in the hottest month (January), and mean monthly minimum of 3.9°C in the coolest month (July) (Bureau of Meteorology, 2018b). Total rainfall was 564 mm in the year prior to sampling (Bureau of Meteorology, 2018a; **Supplementary Figure 1**). Sampling sites at the Boddington location included unamended tailings, rehabilitated tailings, and reference soils. ‘Unamended’ tailings samples were collected from five sites within the active tailings storage area ranging in age from 0 (fresh) – 12 months (1 year) after deposition. ‘Rehabilitated’ tailings samples were collected from a trial site within the tailings storage facility which received 10 cm of topsoil incorporated into the upper 20 cm of tailings. Rehabilitation works occurred in 1999, using undisturbed soil adjacent to the tailings facility. ‘Reference’ soil samples were collected from a site hosting undisturbed soils adjacent to the tailings facility, hosting a mixed native vegetation cover of *Eucalyptus marginata* and *Corymbia calophylla* forest. Samples were collected aseptically in March 2015 with six replicates collected for each site, three of which were stored frozen (microbiological samples, -20°C) and three of which were stored under refrigerated conditions (geochemical samples, 4°C) immediately after collection until analysis.

### Microbial DNA Extraction From Field-Weathered Tailings, Sequencing, Real-Time PCR, and Statistical Analyses

Microbial DNA was extracted from frozen samples (0.60 g tailings or soil) with a MoBio PowerSoil DNA Isolation kit, amplified by PCR with the universal Bacteria/Archaea 16S rRNA primers 926F/1392R (V6–V8 region) and sequenced with the Illumina MiSeq platform according to the procedure outlined in Santini et al. (2016). Sequence data were processed using QIIME according to Santini et al. (2015b) for quality control through to taxonomy assignment. These data have been submitted to the NCBI Sequence Archive under BioProject number PRJNA488346. Samples were rarefied to a uniform depth of 18500 reads per sample. Minimum reads observed per sample before rarefaction was 18531. Relative abundances of OTUs were corrected for differences in 16S rRNA gene copy number using CopyRighter (v 0.46; Angly et al., 2014). 16S rRNA gene copy numbers were used to estimate microbial biomass *via* real-time quantitative PCR, using sample-specific estimates of gene copy number calculated by Copyrighter. DNA was extracted with PowerSoil DNA extraction kit from a known mass of each tailings sample following the protocol outlined above, and analyzed in triplicate with real-time PCR. Prior to running samples, PCR amplification inhibition was tested for five point dilution curves of representative samples with low, medium, and high DNA concentrations. All extracted DNA samples were then diluted from 4 to 100 times with sterile DNA-free water, based on the inhibition control curves and the DNA concentrations as determined by fluorometry (Qubit dsDNA High Sensitivity Kit, Life Technologies). A standard curve was produced by twofold dilutions of genomic DNA from *Escherichia coli* strain B (D4889; Sigma Aldrich). Each PCR reaction had a total reaction volume of 10 μL: 4 μL of template DNA solution, 5 μL SYBR Green Master Mix (Life Technologies), 0.4 μL of 10 μM solutions of the primers 1406F (5′-GYACWCACCGCCCGT-3′) and 1525R (5′-AAGGAGGTGWTCCARCC-3′), and 0.2 μL nuclease-free water. The real time PCR reaction was run with a ViiA 7 Real-Time PCR System and QuantStudio Real-Time PCR software (Applied Biosystems) with the following program conditions: an initial holding phase at 95°C for 20 s followed by 40 cycles of 95°C for 10 s, then 60°C for 20 s. A melt curve was produced by running one cycle at 95°C for 15 s, 60°C for 1 min and then a final cycle of 95°C for 15 s. The cycle threshold (Ct) values were recorded and used to determine the 16S rRNA copy number. For each sample, a mean and standard error of the mean 16S rRNA copy number was determined from the triplicate real-time PCR runs. Detection limit was calculated from standard curves as 1 × 10^5^ cells/g tailings (= 2 × 10^5^ 16S rRNA copies/g tailings, based on the average observed 16S rRNA gene copy number across all communities in our study, calculated by Copyrighter), equating to 25 cells/μL of extracted DNA. Statistically significant differences were identified using ANOVA and Tukey’s HSD as a *post hoc* test for separation of means.

Shannon (H′), reciprocal Simpson, Simpson’s evenness, and Faith’s Phylogenetic Diversity (Faith’s PD) metrics were used to compare alpha diversity between samples; statistically significant differences were identified using ANOVA and Tukey’s HSD as a *post hoc* test for separation of means. Community composition in each sample was visualized by non-metric multidimensional scaling (NMDS) based on Bray–Curtis dissimilarities. PERMANOVA and PERMDISP (Anderson, 2001; Anderson et al., 2006), implemented in PRIMER (v 7.0.10, PRIMER-E, Plymouth, United Kingdom; Clarke and Gorley, 2015), were used to test for statistically significant differences in community composition and dispersion between treatments based on Bray–Curtis distance matrices, with permutations of residuals under a reduced model using 9999 permutations. Permutation *p*-values were used unless low unique permutations necessitated the use of Monte Carlo asymptotic *p*-values. Significant environmental drivers of community composition were identified using DistLM (Anderson, 2002), implemented in PRIMER, with a forward selection procedure using 9999 permutations and Bayesian Information Criterion selection criterion, for model parsimony. Key OTUs accounting for the majority of variation between communities were identified using SIMPER (Clarke, 1993) based on Bray–Curtis dissimilarity matrices, implemented in PRIMER.

### Geochemical and Physical Characterization of Field-Weathered Tailings, and Statistical Analyses

Moisture content was determined by oven-drying at 40°C to constant weight; all further geochemical analyses were performed on oven-dried samples. pH and EC (as a measure of salinity) were determined in a 1:5 soil to water extract (Santini et al., 2013a). Total element concentrations were determined by mixed acid microwave digest (HNO_3_-HCl-HF) followed by ICP-OES analysis of dissolved samples (Rayment and Lyons, 2011). Total N and C were determined by dry combustion (LECO CNS-2000; LECO Corporation, St. Joseph, MI, United States); inorganic C concentration was calculated by difference between total C and residual (organic) C remaining in samples after acid treatment (Rayment and Lyons, 2011; Santini et al., 2013b). Pore water elemental concentrations were determined by equilibrating dried samples with MilliQ water at a 1:1 ratio for 24 h, before filtering to <0.45 μm and determining element concentrations in the extracts by ICP-OES. This was necessary given that most samples (before drying) were too dry to yield pore water by standard centrifugation or filtration approaches. One-way ANOVAs were performed on chemical and physical properties for all sites using a significance level of α = 0.05 (Genstat Release 12.1; VSN International), with Tukey’s honestly significant difference (HSD) (α = 0.05) as a *post hoc* test to separate means if required. If necessary, data were transformed with a natural logarithm or square root prior to ANOVA to meet the assumption of homoscedasticity.

### Dust–Borne Dispersal Simulation Experiment

To test both the survival of microbial cells delivered by dust into the tailings area, and determine the minimum cell input required to trigger growth of the microbial community, a laboratory-based dust addition experiment was performed. Microbial cells separated from the reference soil samples (see Supplementary Information for full details of separation procedure) were added to unamended and rehabilitated tailings from all sampling sites and an autoclaved pure quartz sand (as a geochemically and microbiologically inert control) and incubated at room temperature, in triplicate, for 7 days. Cell separation from the soil was necessary to allow precise addition of cells to the incubations, and to avoid introducing unwanted geochemical variation from the soil matrix. Two addition rates were used: (a) 1.2 × 10^5^ cells/g tailings, to approximate the natural rate of cell addition by dust in the local environment over the first 6 months after deposition; and (b) 2.5 × 10^6^ cells/g tailings, to approximate the natural rate of cell addition by dust in the local environment over the first year after deposition, with an order of magnitude higher dust cell loading rate (see Supplementary Information for further information). Cell counts were monitored during incubations by fluorescence microscopy. A small subsample of tailings was removed and 1 part tailings was mixed with four parts of 0.85% sterile saline water to create a slurry. Cells were stained with a LIVE/DEAD BacLight Bacterial Viability Kit (Invitrogen), according to the manufacturer’s instructions. Stained samples were vortexed and then 1 μl of slurry was pipetted onto a Petroff Hauser counting chamber. Microbial counts of each sample were conducted in triplicate.

## Results

### Microbial Community Composition, Diversity, and Biomass in Field-Weathered Tailings

Microbial community composition in the gold tailings overall was similar to that observed in other alkaline tailings, dominated by Proteobacteria, Actinobacteria, and Firmicutes (Santini and Banning, 2016). Reference soil hosted a greater species richness than unamended tailings, and compositionally, was similar to other studies of natural soils in which Planctomycetes and Acidobacteria as well as Proteobacteria and Actinobacteria tend to dominate community composition (Janssen, 2006; Lauber et al., 2009). The gold tailings tended toward a circumneutral pH over time, similar to that of reference soils, but remained poor in organic C and total N. Salinity was variable over time, increasing early after deposition and then decreasing after 6 months.

**FIGURE 1:**
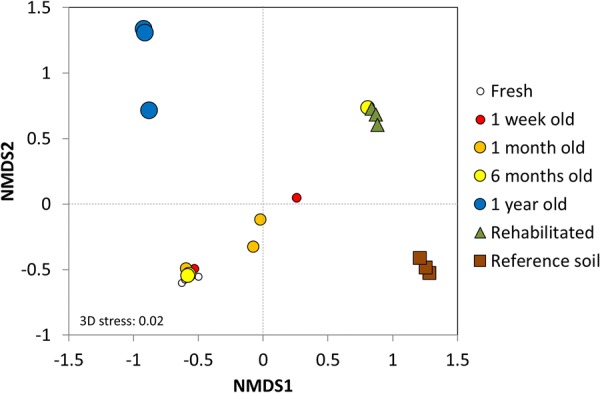
Microbial community composition in unamended field-weathered tailings of various ages (fresh to 1 year old, colored circle markers) after deposition, and rehabilitation (green triangle markers), and reference soils (brown square markers), based on Bray–Curtis dissimilarities analyzed by non-metric multidimensional scaling (NMDS). Three replicates from each site are shown.

Microbial community composition, diversity, and biomass in unamended field-weathered tailings did not significantly change over the first 6 months after deposition, requiring at least 1 year (presumably through dust-based dispersal and recruitment) before significant changes were observed. Beta diversity analyses based on Bray–Curtis dissimilarity matrices revealed that microbial community composition in unamended tailings was invariant over the first 6 months after deposition (**Figure 1**; PERMANOVA: *p* > 0.05, **Supplementary Table 1**; all pairwise comparisons between sites *p* > 0.05, **Supplementary Table 2**); and that these communities were distinctly different to those found in unamended tailings 1 year after deposition, rehabilitated tailings, and reference soils (PERMANOVA: *p* < 0.05, **Supplementary Table 1**; all pairwise comparisons between unamended tailings ≤ 6 months old and other sites < 0.05, **Supplementary Table 2** and **Figure 1**). Further, microbial communities in 1 year old unamended tailings, rehabilitated tailings, and reference soils were all significantly different from each other (PERMANOVA: *p* < 0.05, **Supplementary Table 1**; all pairwise comparisons between sites < 0.05, **Supplementary Table 2** and **Figure 1**). PERMDISP confirmed that differences in community structure between sites were due to compositional changes rather than within-site dispersion (all pairwise comparisons between sites *p* > 0.05; **Supplementary Table 3**).

Microbial biomass in unamended tailings, as estimated by 16S rRNA gene copy numbers, was below detection limits (1 × 10^5^ cells/g) during the first 6 months after deposition, and increased after rehabilitation. Microbial communities in unamended tailings remained significantly lower in species richness and alpha diversity compared to rehabilitated tailings and reference soil during the first year after deposition (**Table 1**). Simpson’s evenness was not significantly different between sites (**Table 1**), indicating that although diversity and richness metrics in rehabilitated tailings and reference soils were higher than those of unamended tailings, diversity metrics were not suppressed in unamended tailings by the dominance of a few key OTUs. Incorporating phylogenetic distance into alpha diversity metrics using Faith’s PD revealed that tailings communities in 1 year old and rehabilitated tailings comprised greater phylogenetic diversity than reference soil communities (**Table 1**).

**Table 1:** Biomass and alpha diversity metrics for microbial communities in tailings and soils.

Site	Microbial biomass (10^6^ cells/g tailings)	Species richness	Shannon	Reciprocal Simpson	Simpson’s evenness	Faith’s PD
Fresh	<0.15	300 ± 101a	4.54 ± 0.57a	11.03 ± 2.11a	0.041 ± 0.007a	0.708 ± 0.002ab
1 week old	< 0.15	837 ± 507a	6.11 ± 1.33ab	36.96 ± 26.1a	0.038 ± 0.005a	0.816 ± 0.103ab
1 month old	< 0.15	834 ± 270a	5.51 ± 0.77ab	14.65 ± 6.67a	0.022 ± 0.009a	0.876 ± 0.079ab
6 months old	< 0.15	1394 ± 1090a	6.07 ± 1.75ab	67.23 ± 57.4ab	0.039 ± 0.006a	1.026 ± 0.285abc
1 year old	0.29 ± 0.07a	382 ± 28a	4.73 ± 0.16a	12.81 ± 2.32a	0.033 ± 0.004a	1.505 ± 0.121bc
Rehabilitated tailings	7.44 ± 3.07a	4027 ± 177b	9.88 ± 0.14bc	225.1 ± 25.6b	0.056 ± 0.005a	1.859 ± 0.326c
Reference soil	105 ± 43.9a	6248 ± 79b	10.9 ± 0.04c	398.7 ± 60.2c	0.064 ± 0.010a	0.449 ± 0.007a

*Values displayed are the mean of three replicates ± 1 standard error of the mean. Sites marked with the same lower case letter in individual columns are not significantly different according to one-way ANOVA. Tukey’s HSD was used to separate means. Microbial (bacterial and archaeal only) biomass was estimated from 16S rRNA copy numbers, using Copyrighter, and with a detection limit of 1.5 × 10^5^ cells/g tailings (= 3 × 10^5^ 16S rRNA copies/g tailings, using the study-wide average of 2 16S rRNA copies per cell).*

**FIGURE 2:**
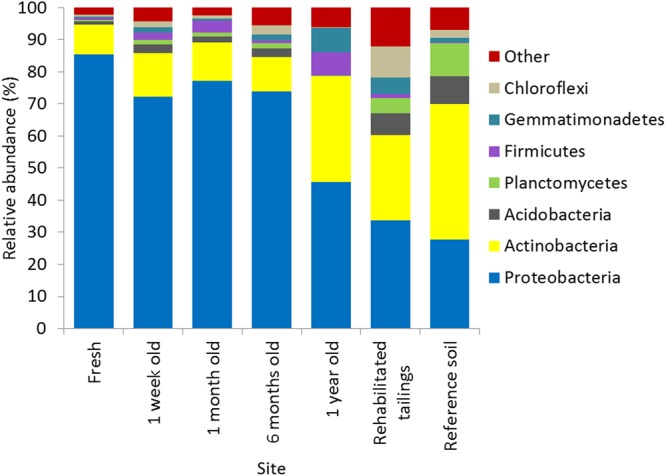
Relative abundance (as a percentage of total sequence reads) of microbial phyla across sites. Values displayed are the mean of three replicates within each site. Phyla present in more than one site at a mean relative abundance of ≥2% are displayed individually; other phyla are summed together as ‘Other.’

Recruitment of novel taxa, particularly those known to host salt and alkali-tolerant lineages, drove the observed shifts in microbial community diversity and biomass in unamended tailings between 6 months and 1 year after deposition. Microbial communities in unamended gold tailings ≤6 months old were dominated by Proteobacteria (particularly Alpha- and Beta-proteobacteria, 45–85% relative abundance) and Actinobacteria (10–30% relative abundance; **Figure 2**). This composition is distinct from those of other alkaline, saline tailings where microbial communities are typically dominated by Gammaproteobacteria and Firmicutes (Santini et al., 2015b). Dust-based recruitment favored Actinobacteria and Firmicutes, as shown by the increase in relative abundance of these phyla in the 1 year old unamended tailings. Rehabilitation supported recruitment of members of a number of common soil phyla including Acidobacteria, Planctomycetes, and Chloroflexi (Lauber et al., 2009; Crowther et al., 2014; Fierer, 2017).

At lower taxonomic ranks, SIMPER analysis identified the major OTUs responsible for differentiating the composition of bacterial communities in unamended tailings ≤ 6 months old, 1 year old unamended tailings, rehabilitated tailings, and reference soils (**Supplementary Table 4**). Bacterial communities in tailings ≤ 6 months old were dominated by members of the *Comamonadaceae* (unclassified genus), *Sphingomonas*, and *Stenotrophomonas* (**Supplementary Table 4**), suggesting that these species were the first colonizers of the tailings due to their dominance during early stages of microbial community succession. The relative abundance of the *Sphingomonas, Comamonadaceae*, and *Stenotrophomonas* OTUs were all significantly positively correlated with each other (all *r* ≥ 0.91; all *p* < 0.001; **Supplementary Table 5**), declining in relative abundance with time and suggesting that they exhibited a coherent response to changing environmental factors (e.g., decreasing pH) during tailings weathering. SIMPER analysis identified recruitment of OTUs from the Clostridiales (unclassified genus) and *Nitriliruptoraceae* (unclassified genus) as major contributors to the dissimilarity between communities present in tailings ≤ 6 months old, and 1 year old tailings (**Supplementary Table 4**), indicating recruitment of likely salt- and alkali-tolerant species through dust-based dispersal. Microbial communities in rehabilitated tailings and reference soils differed not only in alpha diversity from those of unamended tailings, but were also dominated by different OTUs. SIMPER analysis identified representatives of the *Kaistobacter*, Acidimicrobiales (unclassified genus), Acidobacteria (unclassified genus), *Luteimonas*, and *Rhodoplanes* as dominating rehabilitated tailings communities (**Supplementary Table 4**). Each ‘dominant’ OTU in the rehabilitated tailings site was present at ≤ 7% relative abundance, lower than the ≤ 20% relative abundance of ‘dominant’ OTUs in unamended tailings, which reflects the higher alpha diversity in the rehabilitated tailings community (**Table 2**). The dominant OTUs in reference soil communities differed again to those of rehabilitated tailings (**Supplementary Table 4**) and comprised a number of members of the Actinobacteria (*Pseudonocardiaceae* [unclassified genus], Solirubrobacterales [unclassified genus], and *Mycobacterium*), another common and dominant soil phylum (Janssen, 2006).

**Table 2:** pH, EC, and moisture content in tailings and soil samples.

Site	pH	EC	Moisture content	Total C	Inorganic C	Organic C	Total N
		mS cm^-1^	wt %	wt %	wt %	wt %	wt %
Fresh	9.0 ± 0.1c	0.93 ± 0.06ab	27.3 ± 2.87c	0.08 ± 0.01a	0.08 ± 0.02a	0.01 ± 0.003a	0.02 ± 0.005a
1 week old	8.1 ± 0.02b	3.84 ± 0.13c	28.0 ± 2.15c	0.04 ± 0.02a	0.01 ± 0.01a	0.04 ± 0.01a	0.02 ± 0.01a
1 month old	8.1 ± 0.06b	3.88 ± 0.60c	23.0 ± 0.50c	0.06 ± 0.01a	0.02 ± 0.01a	0.03 ± 0.003a	0.03 ± 0.01a
6 months old	8.0 ± 0.1b	4.53 ± 0.71c	0.14 ± 0.01a	0.06 ± 0.01a	0.03 ± 0.02a	0.03 ± 0.01a	0.02 ± 0.002a
1 year old	7.9 ± 0.07b	2.71 ± 0.46bc	3.17 ± 0.13ab	0.05 ± 0.01a	0.03 ± 0.02a	0.02 ± 0.02a	0.02 ± 0.004a
Rehabilitated tailings	6.7 ± 0.05a	0.46 ± 0.07a	6.99 ± 0.28b	0.63 ± 0.14a	–	0.89 ± 0.21a	0.02 ± 0.01a
Reference soil	6.6 ± 0.2a	0.31 ± 0.26a	1.95 ± 0.12ab	8.62 ± 1.37b	2.29 ± 1.16b	6.34 ± 0.69b	0.30 ± 0.04b

*Values displayed are means ± 1 standard error of the mean. “-” indicates concentration below detection limit. Sites marked with the same lower case letter in individual columns are not significantly different according to one-way ANOVA. Tukey’s HSD was used to separate means.*

### Geochemical Properties of Tailings and Soils Shaping Microbial Communities

pH, pore water Mg, and pore water Fe concentrations were identified as having significant relationships with microbial community composition by distance-based linear modeling (DistLM), and cumulatively accounted for 58% of variation in bacterial community composition across sites (**Table 3** and **Supplementary Figure 2**).

**Table 3:** Results of distance-based multivariate multiple regression based on Bray–Curtis dissimilarities for microbial community structures and measured environmental characteristics, using 9999 permutations under a forward selection procedure.

Environmental variable	*p-*Value	Cumulative percentage of variation explained (%)	Multiple partial correlations with dbRDA axes	
			Axis 1	Axis 2
			(46.09% fitted, 26.62% total)	(30.26% fitted, 17.48% total)
pH	0.0001	25.51	0.657	–0.108
Pore water Mg^2+^	0.0005	43.06	–0.078	–0.994
Pore water Fe	0.0001	57.76	–0.750	0.008

*Percentages listed with dbRDA axes indicate the percentage of variation explained by each axis out of the fitted model and total variation.*

In unamended tailings, pH significantly decreased with time after deposition, from initial values of pH 9 to 7.9 after 1 year (**Table 2**). Reference soils had a significantly lower pH (pH 6.55) than all unamended tailings. The addition of topsoil as part of rehabilitation works decreased pH in tailings to values similar to those of reference soils (pH 6.6–6.7).

Although other environmental variables (moisture content, salinity, total element concentrations) were significantly different between sampling sites, these were not significantly related to changes in microbial community composition. Moisture content in unamended tailings decreased over time (27 wt % in fresh tailings to 3 wt % in 1 year old tailings), whereas salinity (measured by EC) increased (0.9 mS cm^-1^ in fresh tailings to 4.5 mS cm^-1^ in 6 month old tailings; **Table 1**). Total N, total C, and (in-)organic C were all significantly higher in reference soil than unamended or rehabilitated tailings (**Table 2**). Rehabilitation works were successful in correcting pH, EC, and moisture content to values similar to those of reference soils, but total and organic C and total N remained the same as in unamended tailings (**Table 2**).

Weathering of the unamended tailings significantly decreased concentrations of Ag, Na, Ca, and Sr (**Supplementary Table 6**), likely through slow dissolution of sparingly soluble minerals after deposition and leaching of metal-rich pore waters. Peaks in pore water Na, Ca, and Sr were observed in the 1 month and 6 months old tailings (**Supplementary Table 7**), which supports mineral dissolution and leaching as a pathway for export of Na, Ca, and Sr in solids. Pore water Al, Fe, Mn, and Si were all significantly higher in reference soils than unamended or rehabilitated tailings samples, which likely reflects the high concentrations of Al, Fe, Mn, and Si oxides in the natural soil matrix (Anand and Paine, 2002; **Supplementary Table 7**). Pore water concentrations of Ca, Mg, K, Na, S, and Sr were all significantly higher in aged unamended tailings than fresh tailings, indicating release through mineral weathering and/or desorption (**Supplementary Table 7**). The addition of local undisturbed soil during rehabilitation elevated Cr concentrations, based on the high Cr concentrations in reference soil, and the significant increase in Cr in rehabilitated tailings compared to 1 year old tailings (**Supplementary Table 6**).

### Laboratory Dust-Borne Dispersal Simulation by Microbial Cell Addition

**FIGURE 3:**
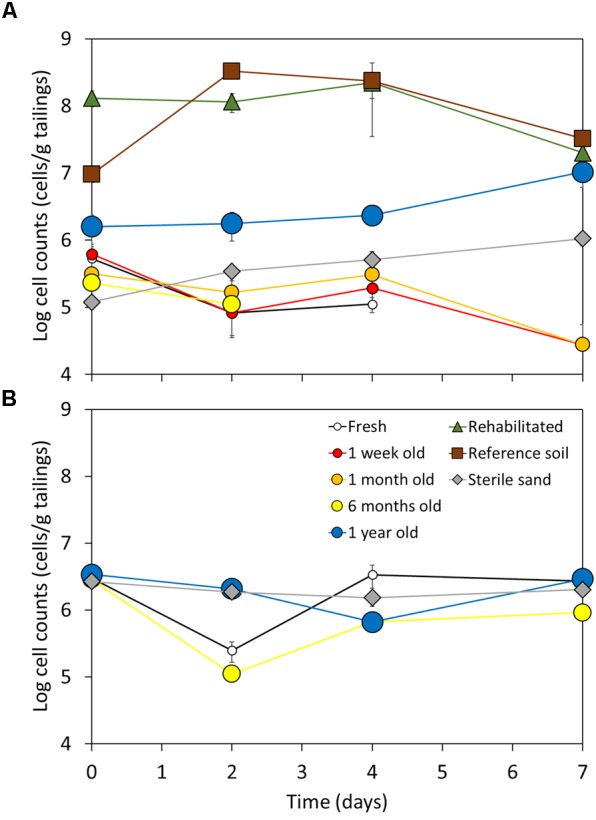
Live cell counts (log transformed) in tailings of various ages (fresh to 1 year old, colored circle markers) after deposition, and rehabilitated (green triangle markers), reference soils (brown square markers), and a sterile, geochemically inert control (autoclaved sand, grey diamond markers) as determined by staining and enumeration by fluorescence microscopy, after addition of microbial cells at **(A)** 1.2 × 10^5^ cells/g sample; or **(B)** 2.5 × 10^6^ cells/g sample. Added cells were extracted from reference soil as described in Methods, to mimic likely microbial community composition on incoming dust. Values shown are the mean of three replicates ± 1 standard error of the mean. Detection limit for live/dead fluorescence microscopy was determined as 8 × 10^4^ cell/g tailings.

Addition of cells at the low (1.2 × 10^5^ cells/g; estimated 6 months’ equivalent) dust addition rate supported biomass maintenance or growth in reference soil, rehabilitated tailings, and 1 year old tailings under laboratory incubation conditions, but was insufficient in unamended tailings less than 1 year old. In fresh, 1 week old, 1 month old, and 6 months old tailings, cell counts decreased well-below initial values over the 7 day incubation, indicating death of added live cells (**Figure 3A**). Given that the unamended tailings less than 1 year old all responded similarly to the low addition rate, only the fresh and 6 months old site samples were re-tested at the higher dust addition rate (2.5 × 10^6^ cells/g; estimated 1 year equivalent dust addition at a higher dust cell loading; **Figure 3B**), which was expected to be sufficient to support maintenance of cell numbers, or even biomass growth. Reference soil and rehabilitated tailings were not re-tested at the higher dust addition rate as they showed a positive response to the low addition rate. Microbial biomass was maintained in all tailings samples after cell addition at the higher rate of 2.5 × 10^6^ cells/g tailings (**Figure 3B**), indicating survival of added cells when a critical minimum cell input was met.

## Discussion

### Both Environmental Conditions and Rates of Dust-Based Dispersal Retard Microbial Community Succession in Tailings

This is the first study to evaluate primary succession in microbial communities in unamended tailings, as an example of an engineered primary successional environment. Rates of microbial community succession in tailings were substantially slower than those of natural environments (e.g., post-wildfire soils, 4–16 weeks; Ferrenberg et al., 2013), with no significant changes occurring until after at least 6 months after deposition. Retarded primary succession in microbial communities could be attributed to dispersal limitation due to physical isolation of tailings storage areas and/or selection pressure imposed by the extreme geochemical and physical conditions of tailings. Our data indicate that both play a role in shaping microbial community successional processes, with dust-based microbial cell additions under both field and laboratory conditions requiring approximately 1 year of dust addition to trigger increases in microbial biomass (**Figures 2, 3**), and environmental factors posing strong selection pressures on microbial propagules arriving by dust-based dispersal, accounting for 58% of variation in microbial community composition (**Table 3** and **Supplementary Figure 2**) and therefore likely retarding microbial community succession.

During the first 6 months after deposition, microbial community composition did not significantly change despite significant decreases in pH (9.0 to 8.0) and moisture content (28.0 to 0.1% wt), and increases in salinity (0.9 to 4.5 mS cm^-1^). Between 6 months and 1 year after deposition, geochemical and physical properties such as pH (7.9–8.0), moisture content (0.1–3.2% wt), and salinity (2.7–4.5 mS cm^-1^) stabilized at lower values (**Table 2**), and microbial community composition significantly changed (**Figure 1** and **Supplementary Table 1**). This suggests geochemical thresholds (e.g., pH 8) above which microbial community succession is substantially retarded, particularly with a low cell input rate from dust only. This is further supported by examining: (a) the environmental tolerances of primary colonizers; and (b) microbial biomass response to dust-borne dispersal. Recruitment of OTUs from bacterial taxa known to host haloalkalitolerant members (Clostridiales, *Nitriliruptoraceae*) drove the shift in microbial community composition between 6 months and 1 year after deposition (**Supplementary Table 4**). Assuming that these OTUs have no natural advantage over other species in terms of dispersal (e.g., smaller cell size, symbiotic relationship with meso-/macro-faunal agents of dispersal), this successional behavior indicates that selection based on tolerance of environmental conditions plays a key role in shaping the microbial community by filtering out those propagules from species incapable of surviving in the tailings.

The observed successional behavior, favoring salt- and alkali-tolerant species in the unamended tailings, does not eliminate a role for slow dispersal rates, and the composition/tolerances of microbial communities arriving through dispersal, in controlling rates of microbial community succession in tailings. The breadth of known environmental tolerances of microorganisms, particularly to high pH, salinity, and moisture conditions, far exceed those presented in the tailings studied here. Therefore, slow dispersal or low abundance in dispersal agents are the only obvious explanations for why known extremophilic species are not seen earlier in the assembly trajectories of the gold tailings communities. Our laboratory-based dust-borne dispersal experiment showed that a high cell addition rate, equivalent to an estimated 1 year of dust input, was required before microbial community biomass could be maintained in the unamended tailings. This matched well with field data indicating that microbial community biomass remained very low (<10^5^ cells/g tailings) until at least 1 year after tailings deposition, and that microbial community composition only showed significant change at 1 year after tailings deposition. At a lower cell addition rate (equivalent to 6 months’ dust input), the role of geochemical thresholds in governing survival of added microbial cells was prominent, with substantial declines in biomass occurring in unamended tailings, but not in 1 year old tailings, rehabilitated tailings, or reference soil. We propose that the majority of microbial cells are likely to either die or enter dormancy on arrival in the fresh tailings environment in response to the unfavorable geochemical conditions, with quorum sensing used to enter and exit dormancy in response to both changing geochemical conditions and changes in microbial biomass and community composition and function. Dormancy and death may be avoided if geochemical conditions are appropriate (e.g., at later times after tailings deposition), or if sufficient biomass and functional capacity exists within the microbial community to offset the energetic costs of maintaining cell homeostasis and metabolism despite unfavorable geochemical conditions (e.g., when large numbers of cells are added by dust, either cumulatively or in major dust deposition events). Overall, our field and laboratory studies provide mutual support for microbial community succession in tailings being limited by both slow rates of dust-borne dispersal as well as subsequent filtering of dust-borne inocula based on environmental tolerances. Quantifying microbial dispersal rates *via* dust and dust inoculum composition in engineered environments such as tailings storage areas in comparison to those of natural primary successional environments, and probing survival and dormancy in dust-borne microbial inocula should be pursued further in a future study.

### Targeted Addition of Soil Inocula During Rehabilitation Shifts the Microbial Community Successional Trajectory in Unamended Tailings Toward That of Natural Soils

Microbial community composition shifted from being dominated by putative autotrophs to (salt- and alkali-tolerant) heterotrophs during primary succession in unamended tailings, and to (mesophilic) heterotrophs in response to rehabilitation. Despite these shifts, unamended tailings, rehabilitated tailings, and reference soils all remained compositionally distinct. Microbial community diversity and richness remained significantly lower in unamended tailings than rehabilitated tailings and reference soil over the first year after deposition, despite significant compositional changes reflecting the role of dust-borne dispersal in shaping microbial community succession. Shannon diversity of the unamended tailings (H′: 4.5–6.1) was similar to other unamended alkaline, saline tailings (bauxite residue H′: 3.2–6.7; Santini et al., 2015b; chromite ore processing residue H′: 0.8–2.3; Brito et al., 2013; uranium mill tailings H′: 3.5–4.0; Dhal and Sar, 2014). As described above, recruitment of new species to unamended tailings was limited by slow dust dispersal rates and subsequently filtered by environmental tolerances. When recruitment to unamended tailings occurred, new OTUs were frequently members of putative or known haloalkalitolerant lineages, causing microbial community composition during succession in unamended tailings to diverge from that of rehabilitated tailings or natural soils. Dominant OTUs in unamended tailings communities ≤ 6 months old (*Comamonadaceae* [unclassified genus], *Sphingomonas*, and *Stenotrophomonas*) are all commonly found in contaminated soils, sediments, and tailings (Macur et al., 2001; Leys et al., 2004; Nemergut et al., 2004; Bondici et al., 2013; Chen et al., 2013; Ozer et al., 2013; Saidi-Mehrabad et al., 2013; Siddique et al., 2014), where they perform a variety of functions related to metal and organic carbon cycling. In these environments, representatives of *Sphingomonas* have been implicated in: degradation of complex hydrocarbons including polycyclic aromatic hydrocarbons (Leys et al., 2004; Liu et al., 2004; Viñas et al., 2005), and iron and arsenic reduction (Macur et al., 2001; Bondici et al., 2013); *Comamonadaceae* have been implicated in: arsenic and antimony oxidation (Terry et al., 2015), degradation of organic compounds including toluene (Sun and Cupples, 2012), and nitrate reduction (Gihring et al., 2011; Wrighton et al., 2014); and *Stenotrophomonas* have been implicated in: arsenic and antimony oxidation (Hamamura et al., 2013), and selenium reduction (Dungan et al., 2003; Siddique et al., 2007). Their co-occurrence in unamended tailings suggests community metabolisms dominated by autotrophy, possibly supplemented by minor heterotrophic activity based on degradation of complex, recalcitrant, or commonly toxic organic carbon sources residual from the gold extraction process (e.g., thiocyanate). The relative abundances of these OTUs were significantly correlated with each other and given their dominance in initial microbial communities, suggests that they were introduced to the tailings during discharge from the refinery, perhaps in water used to decrease tailings viscosity during pumping. The increase in phylogeny-based measures of alpha diversity (e.g., Faith’s PD) in tailings over time (and in response to rehabilitation) further supports recruitment of new species distantly related to those in the original communities, and likely filtered by their tolerances for the challenging environmental conditions present in the tailings.

Topsoil addition during rehabilitation works had a double effect, acting as a microbial inoculant and also correcting geochemical properties of tailings. Rehabilitated tailings hosted a more diverse and mesophilic microbial community than unamended tailings. Rehabilitated tailings also hosted higher relative abundances of key OTUs found in reference soil communities than unamended tailings, which shifted microbial community structure closer to that of reference soil. The dominant OTUs in rehabilitated tailings were associated with a variety of natural, circumneutral pH, fresh to saline aquatic and terrestrial environments, in contrast with those found in unamended tailings which were associated with other contaminated or anthropogenic soil and sediment environments. *Kaistobacter*, like many members of the *Sphingomonadaceae*, have been implicated in degradation of complex organic compounds including isoprene (Gray et al., 2015). Members of the Acidimicrobiales include known acidophilic iron oxidizers and neutrophilic heterotrophs (Stackebrandt, 2014). Acidobacteria are common and ubiquitous in soil, often dominating soil microbial communities (Janssen, 2006). Few representatives have been cultured, but Group 6 species were the dominant group cultured from alkaline soil (George et al., 2011). *Luteimonas* sp. are typically aerobic heterotrophs (Lipski and Stackbrandt, 2005) and have been cultured from a range of aquatic or periodically wet and often saline environments including intertidal sediments (Roh et al., 2008; Park et al., 2011; Romanenko et al., 2013), freshwater and seawater (Baik et al., 2008; Chou et al., 2008) and biofilters (Finkmann et al., 2000). *Rhodoplanes* sp. are typically mesophilic photoheterotrophic bacteria found in anoxic zones in freshwater and wastewater environments (Hiraishi and Imhoff, 2005). A switch from autotroph-dominated communities in unamended tailings to heterotroph-dominated communities in rehabilitated tailings is consistent with previous studies of microbial community responses to rehabilitation (Moynahan et al., 2002; Mendez et al., 2007), and supports the dual role of topsoil addition in rehabilitation efforts as both a source of microbial inocula and organic matter.

Reference soil communities were as diverse as rehabilitated tailings according to most metrics except Faith’s PD, indicating that they hosted high species diversity but that these species were closely related to each other; and like the rehabilitated tailings communities, reference soil communities were dominated by heterotrophs. *Pseudonocardiaceae*, Solirubrobacterales, and *Mycobacterium* (all Actinobacteria), all dominant OTUs in reference soil, are widely distributed in soil and water environments and are generally mesophilic heterotrophs, with some halophilic and/or autotrophic members (Labeda and Goodfellow, 2012; Magee and Ward, 2012; Whitman and Suzuki, 2012). Another dominant OTU in reference soil was a member of the *Rhodospirillaceae*, known as anoxic photoheterotrophs (Garrity et al., 2005) occupying similar environmental niches to *Rhodoplanes* spp. found in rehabilitated tailings. A member of the *Isosphaeraceae* (Planctomycetes), heterotrophic aerobes (some tolerant of micro-oxic conditions) often found in peat bogs and wetlands (Kulichevskaya et al., 2016), was also a dominant OTU in reference soil.

Changes in microbial community composition in unamended tailings over time indicated that dispersal alone is insufficient to shift community composition toward that of reference soils. Dispersal-based recruitment was slow, and when it did occur, recruitment of haloalkalitolerant species shifted microbial community composition away from that of reference soils. Rehabilitation increased the similarity of tailings communities to those of reference soils, likely through the combined action of topsoil as an inoculant source and organic carbon source.

### Environmental Influences on Microbial Community Succession

pH, followed by pore water Mg and pore water Fe, emerged as major controls on microbial community composition across sites in this study. These environmental drivers are consistent with previous studies demonstrating that pH is the key driver of soil bacterial community composition in soils with pH 3.5–9 (Fierer and Jackson, 2006; Rousk et al., 2009). Both pH and pore water Mg concentrations likely reflect weathering of the tailings matrix. pH in the unamended tailings decreased and pore water S increased over time, both of which suggest oxidation of residual sulfides from gold ore in the tailings. Pore water Mg concentration increased over time in the unamended tailings, and the proportion of pore water Mg to total Mg increased with tailings age, both of which indicate dissolution and release of Mg^2+^ from Mg-bearing minerals (e.g., dolomite) in the tailings during weathering. Pore water Fe concentration was well-correlated with total Fe concentration (*r*^2^ = 0.914, *p* < 0.01, two-tailed), and higher in both rehabilitated tailings and reference soil than unamended tailings, suggesting that pore water Fe reflects the influence of the natural soil mineral matrix. The relatively low total percentage of variation in bacterial community structure accounted for by environmental variables using DistLM (58%; **Table 3**) further supports a significant role for stochastic processes such as dust-based dispersal and recruitment of alkali- and salt- tolerant species in shaping microbial community composition.

The observed trajectory of microbial community assembly in unamended tailings, and comparisons with communities in rehabilitated tailings and reference soil indicates roles for both environmental factors and slow rates of dust-borne dispersal in shaping primary successional processes in engineered environments. Both field and laboratory experiments revealed that low rates of microbial cell influx into the unamended tailings, primarily by dust-borne dispersal due to physical and spatial isolation from other potential inoculant sources, limited microbial community growth and likely retarded succession. Environmental factors, such as pH, posed strong selection pressures on those inoculants that did arrive on site by dust-borne dispersal, as indicated by the dominance of haloalkalitolerant species in unamended tailings once evidence of succession was observed, between 6 months and 1 year after deposition. Dust-borne dispersal alone was insufficient to shift microbial community composition in unamended tailings toward that of reference soils; and in fact, the strong selection pressures for haloalkalitolerant species caused microbial community composition in tailings to diverge from that of reference soils during dispersal-based recruitment. Accelerating and guiding microbial community assembly and succession in engineered environments therefore requires addition of targeted inocula to introduce species of interest and/or amendment of the geochemical properties of the tailings to encourage microbial community diversification and succession. Topsoil addition during tailings rehabilitation served as both a microbial inoculant and as an agent of chemical and physical amelioration; the combined effect was to shift microbial community composition in tailings toward that of reference soil. Although rehabilitation shifted microbial community composition toward that of reference soils, with both communities dominated by aerobic heterotrophs, the taxonomic identity of dominant species within each site differed. Given that similar environmental niches (and functions) can be occupied (and performed) by different species, e.g., *Rhodoplanes* in rehabilitated tailings and *Rhodospirillaceae* in reference soil, assessment of functional capacity should be coupled with assessment of alpha and beta diversity and taxonomic identity when assessing rehabilitation success against a reference soil community.

## Author Contributions

TS conceptualized the study, coordinated sample collection and analysis, analyzed samples and data, and wrote the manuscript. MR completed sample and data analysis and contributed to manuscript text. JH and JN completed sample and data analysis. The manuscript was written through contributions of all authors. All authors have given approval to the final version of the manuscript.

## Conflict of Interest Statement

The authors declare that the research was conducted in the absence of any commercial or financial relationships that could be construed as a potential conflict of interest.

## Abbreviations

ANOVA: analysis of variance
DistLM: distance-based multivariate multiple regression
DNA: deoxyribonucleic acid
EC: electrical conductivity
OTU: operational taxonomic unit
PERMANOVA: permutational multivariate analysis of variance
PERMDISP: permutational multivariate analysis of dispersion
SIMPER: similarity percentage
