# Supplementary material for: Extreme Geochemical Conditions and Dispersal Limitation Retard Primary Succession of Microbial Communities in Gold Tailings
Source: Front Microbiol. 2018 Nov 28;9:2785. doi: 10.3389/fmicb.2018.02785 (PMC6279923; doi:10.3389/fmicb.2018.02785)
Supplement: Supplementary file 1 [file Data_Sheet_1.docx]

Supplementary Material

**Title:** Extreme geochemical conditions and dispersal limitation retard primary succession of microbial communities in gold tailings

**Authors:** Talitha C. Santini, Maija Raudsepp, Jessica Hamilton, Jasmine Nunn

**Contents:** 2 figures, 7 tables, 16 pages

**Materials and methods**

*Sampling location and sites*

Rainfall in the year prior to sampling was slightly above the long term (~20 year) average of 510 mm, with the majority falling over the typical wet winter period of April to October (Supplementary Information Figure 1).

**Supplementary Information Figure 1**. Monthly rainfall during the year immediately preceding sampling, as recorded at the closest weather station to the sampling site, Wandering, WA (010917) (Bureau of Meteorology, 2018a).

*Dust–borne dispersal simulation experiment*

Cell separation was completed by repeating three rounds of the following: vortexing 5 g reference soil (stored at 4 ℃) with 15 ml sterile saline (0.85 %) solution for 30 seconds, centrifuging at 500 rpm for 10 seconds to settle large particles, transferring supernatant to a fresh 50 mL Falcon tube and then centrifuging at 14000 rpm for 5 minutes to create a cell pellet. The same initial 5 g of reference soil was vortexed with fresh saline multiple times to maximize cell extraction and decrease the amount of organics that were added to the tailings with the pelleted cells. Given that we were testing tailings that had already received dust after deposition in the field, we wanted to avoid introducing any geochemical variation associated with the geochemical or mineralogical composition of the dust into our dispersal simulation experiment, which would not have been representative of field conditions. DNA sequencing of microbial communities in the reference soil before vortexing, after triple vortexing, and in the cell pellet (following procedures outlined above) verified that the separation process did not induce significant changes in microbial community composition between the reference soil and the pellet.

Two addition rates of cells to tailings were used to mimic different dust deposition rates in the environment. The first, 1.2 x 10^5^ cells/g tailings, was designed to approximate the natural rate of cell addition by dust in the local environment over the first six months after deposition; the second, 2.5 x 10^6^ cells/g tailings, was designed to approximate the natural rate of cell addition by dust in the local environment over the first year after deposition, with an order of magnitude higher dust cell loading rate. Addition rates were determined as follows. Given that observed Australian dust deposition rates are typically ≤ 186 mg/m^2^/day (Hingston and Gailitis, 1976; Leys and McTainsh, 1999; Hesse and McTainsh, 2003; Cattle et al., 2009), on the low end of global averages, we selected a generous dust deposition rate of 200 mg/m^2^/day. Assuming a dust cell loading similar to that of soil (1 x 10^7^-1 x 10^9^ cells/g; Richter and Markewitz, 1995; Whitman et al., 1998; Griffin, 2007), at 1 x 10^8^ cells/g, approximately 3.6 x 10^9^ cells/m^2^ tailings would be received over a six month period. Our tailings sampling captured samples from 0-2 cm below surface, with tailings having an estimated bulk density of 1.5 g/cm^3^; hence this rate of cell addition equates to 1.2 x 10^5^ cells/g tailings in the upper 2 cm of tailings over the first six months after tailings deposition.

Given that the added cell concentration at the 1.2 x 10^5^ cells/g tailings addition rate was near the detection limit for enumeration with fluorescence microscopy, and to better observe the response of cells to tailings, incubations were repeated for fresh, 6 month and 1 year old tailings samples, and autoclaved sand, using a higher cell concentration (2.5 x 10^6^ cells/gram of tailings). This 20-fold increase is equivalent to an order of magnitude increase in cell loading on incoming dust (ten-fold increase), and doubling the length of time over which the tailings received dust from six to 12 months (two-fold increase). Given that both increases in microbial biomass and shifts in microbial community composition were observed between the six month and one year old unamended tailings microbial communities, increasing the cell addition rate to this value allowed us to test whether dust-based microbial cell addition rates were limiting microbial community establishment and assembly. We did not test lower rates of microbial cell addition, equivalent to daily, weekly, or monthly addition rates, due to the lack of growth observed at the six months’ equivalent cell addition rate, and methodological challenges associated with cell quantification as described above.

**Supplementary Information Table 1.** PERMANOVA results from analysis of Bray-Curtis dissimilarity matrix, with site as factor. *P*-values are based on 9999 permutations. F values are pseudo-F determined by permutation. R^2^ value can be interpreted as the proportion of variation explained.

| **Source of variation** | **d.f.** | **SS** | ***F*** | **R^2^** | ***P*-value** |
| --- | --- | --- | --- | --- | --- |
| *All sites* | | | | | |
| Site | 6 | 4.7913 | 5.6263 | 0.21887 | 0.0001 |
| Residuals | 14 | 1.9870 |  | 0.14193 |  |
| Totals | 20 | 6.7783 |  |  |  |
| *Non-rehabilitated tailings ≤ 6 months old* | | | | | |
| Site | 3 | 0.5078 | 0.9823 | 0.00102 | 0.3745 |
| Residuals | 8 | 1.3786 |  | 0.17232 |  |
| Totals | 11 | 1.8864 |  |  |  |

**Supplementary Information Table 2.** PERMANOVA results from pairwise comparisons of sites based on Bray-Curtis dissimilarity matrix. Monte-Carlo asymptotic P-values (MC) are based on 9999 permutations. *t* values are for pairwise comparisons between sites.

| **Comparisons (sites)** | ***t*** | ***P*-value (MC)** |
| --- | --- | --- |
| Fresh vs 1 week old | 1.1970 | 0.2758 |
| Fresh vs 1 month old | 1.5286 | 0.1356 |
| Fresh vs 6 months old | 1.0056 | 0.3735 |
| Fresh vs 1 year old | 3.9231 | 0.0032 |
| Fresh vs rehabilitated | 4.5358 | 0.0016 |
| Fresh vs reference soil | 4.6500 | 0.0017 |
| 1 week old vs 1 month old | 0.9399 | 0.4708 |
| 1 week old vs 6 months old | 0.6011 | 0.6994 |
| 1 week old vs 1 year old | 2.6795 | 0.0107 |
| 1 week old vs rehabilitated | 2.5617 | 0.0189 |
| 1 week old vs reference soil | 2.8757 | 0.0093 |
| 1 month old vs 6 months old | 0.9389 | 0.4581 |
| 1 month old vs 1 year old | 2.5803 | 0.0124 |
| 1 month old vs rehabilitated | 2.7215 | 0.0109 |
| 1 month old vs reference soil | 2.7639 | 0.0098 |
| 6 months old vs 1 year old | 2.0482 | 0.0440 |
| 6 months old vs rehabilitated | 1.7330 | 0.1166 |
| 6 months old vs reference soil | 2.1856 | 0.0320 |
| 1 year old vs rehabilitated | 3.3718 | 0.0048 |
| 1 year old vs reference soil | 3.5012 | 0.0026 |
| Rehabilitated vs reference soil | 3.3266 | 0.0038 |

**Supplementary Information Table 3.** PERMDISP results from analysis of Bray-Curtis dissimilarity matrix, with site as group factor. *P*-values are based on 9999 permutations. Pseudo-F (determined by permutation) was 3.4156 with d.f._1_ =6 and d.f._2_ =14. *t* values are for pairwise comparisons between levels of factor (site). Means and S.E. (standard errors) calculated from within-group dispersion.

| **Comparisons** | ***t*** | ***P*-value** |
| --- | --- | --- |
| Fresh vs 1 week old | 2.6854 | 0.1997 |
| Fresh vs 1 month old | 4.4012 | 0.1030 |
| Fresh vs 6 months old | 2.7148 | 0.1024 |
| Fresh vs 1 year old | 4.4127 | 0.0997 |
| Fresh vs rehabilitated | 3.5313 | 0.0987 |
| Fresh vs reference soil | 3.7224 | 0.1022 |
| 1 week old vs 1 month old | 0.2551 | 0.7997 |
| 1 week old vs 6 months old | 0.9439 | 0.4963 |
| 1 week old vs 1 year old | 0.5891 | 1.0000 |
| 1 week old vs rehabilitated | 1.1226 | 0.6010 |
| 1 week old vs reference soil | 1.3059 | 0.7973 |
| 1 month old vs 6 months old | 0.8705 | 0.7992 |
| 1 month old vs 1 year old | 1.3895 | 0.5039 |
| 1 month old vs rehabilitated | 2.3271 | 0.1038 |
| 1 month old vs reference soil | 2.6832 | 0.1049 |
| 6 months old vs 1 year old | 1.4299 | 0.6078 |
| 6 months old vs rehabilitated | 1.7479 | 0.3934 |
| 6 months old vs reference soil | 1.8548 | 0.4048 |
| 1 year old vs rehabilitated | 1.5268 | 0.1959 |
| 1 year old vs reference soil | 2.2333 | 0.0976 |
| Rehabilitated vs reference soil | 0.7446 | 0.3897 |
| **Within-group dispersion** | **Mean** | **S.E.** |
| Fresh | 0.1431 | 0.0266 |
| 1 week old | 0.3208 | 0.0606 |
| 1 month old | 0.3387 | 0.0356 |
| 6 months old | 0.2411 | 0.0077 |
| 1 year old | 0.2515 | 0.0118 |
| Rehabilitated | 0.4343 | 0.1039 |
| Reference soil | 0.2836 | 0.0174 |

**Supplementary Information Table 4.** Relative abundance of dominant OTUs in tailings and soil samples at each site, as identified by SIMPER. Dominant OTUs were defined as the five OTUs in each site with the highest relative abundance, and are highlighted in bold in the table below. ‘Rel Abund’ indicates the means of relative abundance (as a percentage of total sequence reads) in three replicates for each site. ‘% Sim’ indicates the percentage contribution of each OTU to the overall similarity among microbial communities in three replicates at each site. ‘-‘ indicates no sequence reads were recovered for this OTU at a particular site.

| **OTU** | **Fresh** | | **1 week old** | | **1 month old** | | **6 months old** | | **1 year old** | | **Rehabilitated tailings** | | **Reference soil** | |
| --- | --- | --- | --- | --- | --- | --- | --- | --- | --- | --- | --- | --- | --- | --- |
|  | **Rel Abund** | **% Sim** | **Rel Abund** | **% Sim** | **Rel Abund** | **% Sim** | **Rel Abund** | **% Sim** | **Rel Abund** | **% Sim** | **Rel Abund** | **% Sim** | **Rel Abund** | **% Sim** |
| *Sphingomonas* sp. | **20.04** | **23.00** | **11.28** | **13.92** | **9.81** | **15.22** | **11.67** | **16.65** | 1.00 |  | 0.049 |  | 0.67 |  |
| *Comamonadaceae* sp. 1 | **17.03** | **20.26** | **16.03** | **21.59** | **13.02** | **15.73** | **13.01** | **19.31** | 1.75 |  | 0.24 |  | 0.31 |  |
| *Comamonadaceae* sp. 2 | **13.31** | **15.54** | **13.25** | **16.51** | **10.55** | **12.79** | **11.01** | **14.60** | 1.09 |  | 0.094 |  | 0.21 |  |
| *Stenotrophomonas* sp. | **13.10** | **14.14** | **8.17** | **10.68** | **7.01** | **10.56** | **9.29** | **13.43** | 0.52 |  | 0.026 |  | 0.0042 |  |
| *Caulobacter* sp. | **9.08** | **9.67** | **4.78** | **5.12** | 3.39 |  | **5.16** | **6.86** | 0.38 |  | 0.0017 |  | 0.0049 |  |
| *Ectothiorhodospiraceae* sp. | 0.0027 |  | 0.41 |  | **15.32** | **5.47** | 0.13 |  | 0.31 |  | 0.10 |  | 0.0072 |  |
| Actinomycetales sp. | 0.11 |  | 0.34 |  | 0.15 |  | 0.10 |  | **13.12** | **20.70** | 0.32 |  | 0.36 |  |
| *Xanthomonadaceae* sp. | 0.28 |  | 0.33 |  | 0.28 |  | 1.23 |  | **16.50** | **17.88** | 1.87 |  | 0.048 |  |
| *Nitriliruptoraceae* sp. | - |  | 0.052 |  | 0.18 |  | 0.16 |  | **15.48** | **14.91** | 0.59 |  | - |  |
| Clostridiales sp. | 0.090 |  | 0.16 |  | 0.058 |  | 0.030 |  | **5.98** | **10.08** | 0.082 |  | 0.0011 |  |
| Chromatiales sp. | - |  | 0.10 |  | 0.66 |  | 0.33 |  | **8.53** | **7.09** | 0.43 |  | - |  |
| *Kaistobacter* sp. | 0.053 |  | 1.24 |  | 0.54 |  | 2.41 |  | 0.016 |  | **7.33** | **8.38** | 0.38 |  |
| Acidimicrobiales sp. | - |  | 1.64 |  | 0.64 |  | 1.77 |  | 0.077 |  | **5.67** | **7.15** | 0.93 |  |
| Acidobacteria sp. | 0.40 |  | 1.32 |  | 0.53 |  | 1.58 |  | 0.0063 |  | **4.62** | **5.50** | 1.03 |  |
| *Luteimonas* sp. | - |  | 0.66 |  | 0.26 |  | 1.22 |  | 0.0023 |  | **2.97** | **3.15** | - |  |
| *Rhodoplanes* sp. | 0.12 |  | 0.85 |  | 0.37 |  | 0.74 |  | 0.0066 |  | **2.68** | **3.06** | 2.03 |  |
| *Mycobacterium* sp. | 4.75 |  | 3.22 |  | 3.38 |  | 2.92 |  | 0.20 |  | 0.77 |  | **9.85** | **11.04** |
| Solirubrobacterales sp. | 0.67 |  | 1.51 |  | 0.99 |  | 0.68 |  | 0.0026 |  | 2.64 |  | **6.88** | **7.83** |
| *Pseudonocardiaceae* sp. | 0.16 |  | 0.40 |  | 0.19 |  | 0.11 |  | 0.012 |  | 0.49 |  | **5.95** | **3.79** |
| *Isosphaeraceae* sp. | 0.016 |  | 0.11 |  | 0.17 |  | 0.047 |  | 0.0029 |  | 0.039 |  | **3.20** | **3.57** |
| *Rhodospirillaceae* sp. | 0.17 |  | 0.74 |  | 0.46 |  | 0.83 |  | 0.0090 |  | 2.16 |  | **2.98** | **3.13** |

**Supplementary Information Table 5.** Correlation matrix between relative abundances of the four OTUs identified by SIMPER analysis as defining microbial communities in unamended tailings ≤ 6 months old. OTU names correspond to those used in Table 4 of the main paper. Asterisks indicate significance (two-tailed, α=0.05) at: p<0.001 (***); p<0.01 (**); and p<0.05 (*).

| Sphingomonas sp. |  |  |  |
| --- | --- | --- | --- |
| Comamonadaceae sp. 1 | 0.9114*** |  |  |
| Comamonadaceae sp. 2 | 0.9208*** | 0.9903*** |  |
| Stenotrophomonas sp. | 0.9901*** | 0.9388*** | 0.9408*** |
|  | Sphingomonas sp. | Comamonadaceae sp. 1 | Comamonadaceae sp. 2 |


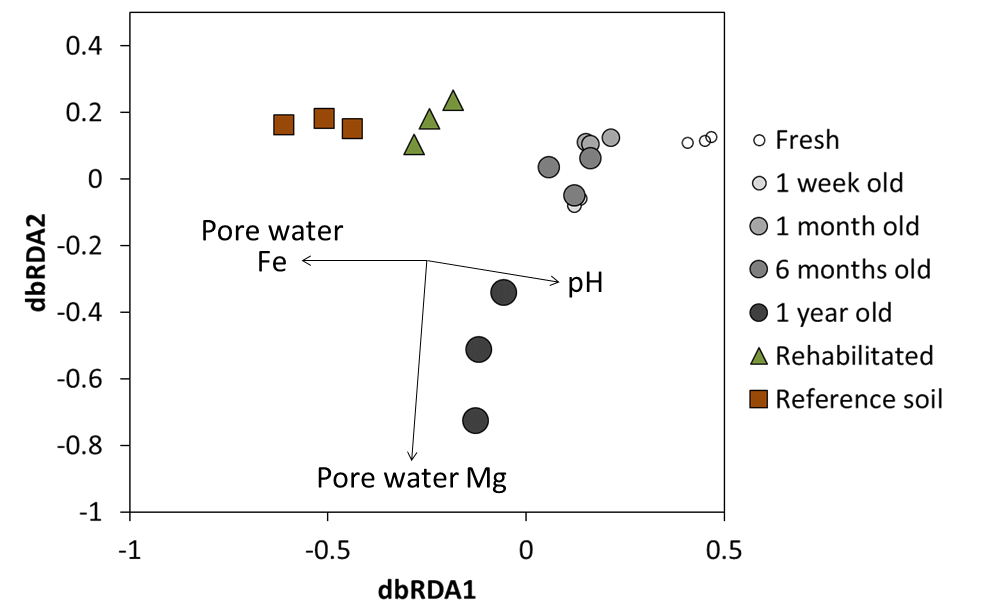


**Supplementary Information Figure 2.** Distance-based redundancy analysis (dbRDA) plot of DistLM results for unamended tailings (fresh to 1 year old), rehabilitated tailings, and reference soil. Only geochemical variables with significant relationships with microbial community composition are shown, against axes 1 (dbRDA1, accounting for 46.1% of fitted variation and 26.6% of total variation) and 2 (dbRDA2, accounting for 30.3 % of fitted variation and 17.5 % of total variation) identified in the analysis. Three replicates are shown for each sampling site.

**Supplementary Information Table 6.** Total element concentrations in tailings and soil samples. Values displayed are means ± 1 standard error of the mean. Sites marked with the same lower case letter in individual columns are not significantly different according to one-way ANOVA. Tukey’s HSD was used to separate means. Results for Au, Co, Mo, Se, and Zn are not shown as concentrations did not significantly differ between sites.

| *Major elements* | | | | | | | | | | | |
| --- | --- | --- | --- | --- | --- | --- | --- | --- | --- | --- | --- |
| **Site** | **Al** | **Ca** | **Fe** | **K** | **Mg** | **Mn** | **Na** | **P** | **S** | **Si** | **Ti** |
|  | **wt %** | **wt %** | **wt %** | **wt %** | **wt %** | **ppm** | **wt %** | **ppm** | **wt %** | **wt %** | **wt %** |
| Fresh | 4.01 ± 0.44 a | 1.56 ± 0.16 bc | 4.49 ± 0.03 a | 2.11 ± 0.08 b | 0.41 ± 0.14 a | 423.7 ± 35.85 a | 2.86 ± 0.12 d | 631.0 ± 33.63 bc | 0.11 ± 0.02 a | 30.3 ± 0.49 bc | 0.36 ± 0.02 a |
| 1 week old | 3.56 ± 0.19 a | 2.09 ± 0.05 c | 3.87 ± 0.09 a | 1.98 ± 0.15 b | 0.35 ± 0.03 a | 419.2 ± 1.36 a | 2.71 ± 0.05 cd | 738.1 ± 20.09 c | 0.26 ± 0.01 ab | 29.6 ± 0.63 bc | 0.39 ± 0.00 a |
| 1 month old | 3.70 ± 0.60 a | 1.89 ± 0.03 bc | 3.26 ± 0.06 a | 1.61 ± 0.04 ab | 0.31 ± 0.02 a | 426.4 ± 13.83 a | 2.58 ± 0.06 cd | 680.4 ± 48.31 c | 0.22 ± 0.05 ab | 30.7 ± 0.26 c | 0.39 ± 0.02 a |
| 6 months old | 6.90 ± 0.51 ab | 2.22 ± 0.11 c | 4.50 ± 0.02 a | 2.00 ± 0.02 b | 1.29 ± 0.18 b | 529.6 ± 13.09 b | 2.54 ± 0.03 cd | 695.3 ± 33.31 c | 0.38 ± 0.10 b | 28.3 ± 0.27 bc | 0.39 ± 0.01 a |
| 1 year old | 3.98 ± 0.60 a | 1.32 ± 0.18 b | 3.52 ± 0.01 a | 1.24 ± 0.03 ab | 0.36 ± 0.11 a | 442.2 ± 19.61 ab | 2.28 ± 0.02 bc | 603.6 ± 23.61 bc | 0.26 ± 0.02 ab | 29.5 ± 0.75 bc | 0.48 ± 0.02 ab |
| Rehabilitated tailings | 6.14 ± 0.62 ab | 1.30 ± 0.25 b | 6.85 ± 1.88 a | 1.03 ± 0.10 ab | 0.41 ± 0.06 a | 402.2 ± 13.11 a | 1.82 ± 0.20 b | 506.9 ± 25.01 bc | 0.15 ± 0.02 a | 26.3 ± 1.66 b | 0.60 ± 0.06 b |
| Reference soil | 8.58 ± 1.35 b | 0.16 ± 0.01 a | 19.6 ± 2.19 b | 0.84 ± 0.56 a | 0.03 ± 0.01 a | 355.3 ± 23.40 a | 0.15 ± 0.09 a | 237.5 ± 45.47 a | 0.065 ± 0.038 a | 14.2 ± 0.99 a | 1.09 ± 0.04 c |
| *Minor elements* | | | | | | | | | | | |
| **Site** | **Ag** | **As** | **Ba** | **Cd** | **Cr** | **Cu** | **Ga** | **Ni** | **Pb** | **Sr** | **V** |
|  | **ppm** | **ppm** | **ppm** | **ppm** | **ppm** | **ppm** | **ppm** | **ppm** | **ppm** | **ppm** | **ppm** |
| Fresh | 101.8 ± 36.25 b | 12.18 ± 0.07 ab | 264.3 ± 83.74 c | 0.33 ± 0.13 a | 62.26 ± 4.89 a | 192.3 ± 6.19 b | 0.90 ± 0.06 a | 24.87 ± 3.24 a | 3.61 ± 1.12 a | 266.5 ± 31.43 c | 85.65 ± 4.84 ab |
| 1 week old | 28.50 ± 6.64 a | 10.74 ± 0.51 ab | 188.5 ± 9.71 abc | 0.26 ± 0.03 a | 93.42 ± 0.93 a | 188.6 ± 5.31 b | 1.01 ± 0.06 ab | 38.34 ± 6.19 ab | 2.54 ± 0.91 a | 218.7 ± 3.87 bc | 90.41 ± 1.07 ab |
| 1 month old | 16.02 ± 1.37 a | 10.71 ± 0.15 ab | 195.7 ± 1.76 abc | 0.26 ± 0.05 a | 70.33 ± 2.55 a | 145.4 ± 6.53 b | 0.87 ± 0.02 a | 27.9 ± 1.07 ab | 2.43 ± 0.84 a | 211.9 ± 10.83 bc | 81.56 ± 3.34 a |
| 6 months old | 14.00 ± 2.79 a | 12.57 ± 0.32 abc | 216.0 ± 12.03 bc | 0.23 ± 0.05 a | 85.43 ± 2.47 a | 136.5 ± 4.09 b | 1.07 ± 0.04 ab | 41.54 ± 1.13 ab | 4.37 ± 0.42 a | 194.2 ± 2.55 b | 96.51 ± 1.72 ab |
| 1 year old | 9.92 ± 2.36 a | 14.83 ± 1.31 bc | 105.0 ± 5.25 abc | 0.17 ± 0.02 a | 70.42 ± 3.54 a | 292.6 ± 25.58 c | 1.01 ± 0.07 ab | 31.59 ± 6.71 ab | 5.26 ± 1.49 ab | 182.7 ± 3.25 b | 98.95 ± 5.42 ab |
| Rehabilitated tailings | 11.92 ± 0.44 a | 17.25 ± 1.71 c | 112.2 ± 10.72 abc | 0.40 ± 0.08 a | 192.7 ± 52.45 b | 336.8 ± 40.09 c | 2.03 ± 0.58 b | 97.39 ± 37.6 b | 3.47 ± 1.41 a | 155.0 ± 18.08 b | 193.6 ± 54.04 b |
| Reference soil | 10.11 ± 0.66 a | 8.54 ± 1.45 a | 39.7 ± 4.52 a | 0.98 ± 0.22 b | 231.9 ± 8.03 b | 7.99 ± 1.33 a | 3.88 ± 0.17 c | 9.62 ± 4.35 a | 11.05 ± 1.70 b | 30.29 ± 3.80 a | 563.2 ± 23.57 c |

**Supplementary Information Table 7.** Pore water (dissolved) concentrations (in ppm) in tailings and soil samples, as determined by water extraction. Values displayed are means ± 1 standard error of the mean. Sites marked with the same lower case letter in individual columns are not significantly different according to one-way ANOVA. Tukey’s HSD was used to separate means. Results for As, Cd, Ni, P, Se, and Zn are not shown as concentrations did not significantly differ between sites.

| *Major elements* |  |  |  |  |  |  |  |  |  |
| --- | --- | --- | --- | --- | --- | --- | --- | --- | --- |
| **Site** | **Al** | **Ca** | **Fe** | **K** | **Mg** | **Na** | **S** | **Si** | **Sr** |
|  | **ppm** | **ppm** | **ppm** | **ppm** | **ppm** | **ppm** | **ppm** | **ppm** | **ppm** |
| Fresh | 0.40 ± 0.10 a | 236.3 ± 12.06 b | 0.18 ± 0.01 a | 67.45 ±  3.74 ab | 18.3 ± 0.75 ab | 561.2 ± 50.87 ab | 259.8 ± 12.43 b | 4.23 ± 0.34 a | 1.31 ± 0.05 a |
| 1 week old | 0.73 ± 0.17 a | 822.6 ± 10.74 cd | 0.16 ± 0.02 a | 191.4 ± 9.150 c | 40.0 ± 1.09 b | 1664 ± 72.34 bc | 716.8 ± 19.13 c | 4.26 ± 0.41 a | 4.43 ± 0.11 bc |
| 1 month old | 0.56 ± 0.01 a | 800.7 ± 17.77 c | 0.19 ± 0.01 a | 205.1 ± 15.00 c | 22.4 ± 0.79 ab | 1770 ± 142.1 bc | 793.3 ± 26.35 c | 4.16 ± 0.16 a | 4.47 ± 0.41 bc |
| 6 months old | 0.59 ± 0.03 a | 953.3 ± 59.01 d | 0.18 ± 0.01 a | 238.0 ± 37.83 c | 32.8 ± 3.39 b | 2833 ± 610.9 c | 843.5 ± 40.76 c | 4.70 ± 0.25 a | 6.02 ± 0.75 c |
| 1 year old | 0.52 ± 0.03 a | 680.6 ± 31.34 c | 0.15 ± 0.01 a | 153.4 ± 25.44 bc | 89.5 ± 11.3 c | 957.5 ± 270.7 ab | 742.7 ± 65.09 c | 3.50 ± 0.39 a | 3.43 ± 0.36 b |
| Rehabilitated tailings | 0.71 ± 0.21 a | 164.6 ± 37.13 b | 0.47 ± 0.10 a | 54.5 ± 0.80 a | 21.8 ± 4.19 ab | 145.2 ± 16.38 a | 251.7 ± 42.38 b | 4.17 ± 0.55 a | 0.57 ± 0.11 a |
| Reference soil | 79.5 ± 7.17 b | 10.12 ± 2.54 a | 46.74 ± 2.21 b | 4.11 ± 0.36 a | 5.14 ± 1.38 a | 10.19 ± 2.46 a | 44.6 ± 2.72 a | 9.49 ± 0.26 b | 0.05 ± 0.01 a |
| *Minor elements* |  |  |  |  |  |  |  |  |  |
| **Site** | **B** | **Ba** | **Co** | **Cr** | **Cu** | **Mn** | **Mo** | **Pb** |  |
|  | **ppm** | **ppm** | **ppm** | **ppm** | **ppm** | **ppm** | **ppm** | **ppm** |  |
| Fresh | 0.08 ± 0.03 a | 0.18 ± 0.01 ab | 0.19 ± 0.05 ab | 0.017 ± 0.004 ab | 0.001 ± 0.001 a | 0.016 ± 0.005 a | 0.051 ± 0.010 a | 0.017 ± 0.006 a |  |
| 1 week old | 0.10 ± 0.02 a | 0.18 ± 0.02 ab | 0.35 ± 0.02 bc | 0.020 ± 0.011 ab | 0.003 ± 0.002 ab | 0.069 ± 0.002 a | 0.073 ± 0.010 a | 0.065 ± 0.014 b |  |
| 1 month old | 0.14 ± 0.02 ab | 0.15 ± 0.04 ab | 0.48 ± 0.05 c | 0.043 ± 0.020 ab | 0.010 ± 0.007 ab | 0.045 ± 0.006 a | 0.072 ± 0.010 a | 0.058 ± 0.011 ab |  |
| 6 months old | 0.12 ± 0.01 a | 0.26 ± 0.05 b | 0.37 ± 0.06 bc | 0.048 ± 0.009 ab | 0.019 ± 0.004 b | 0.025 ± 0.001 a | 0.102 ± 0.018 ab | 0.065 ± 0.006 b |  |
| 1 year old | 0.22 ± 0.02 b | 0.15 ± 0.02 ab | 0.30 ± 0.06 bc | 0.001 ± 0.001 a | 0.007 ± 0.002 ab | 0.080 ± 0.009 a | 0.157 ± 0.016 b | 0.063 ± 0.009 ab |  |
| Rehabilitated tailings | 0.10 ± 0.01 a | 0.15 ± 0.02 ab | 0.20 ± 0.01 ab | 0.036 ± 0.017 ab | 0.005 ± 0.003 ab | 0.283 ± 0.095 a | 0.071 ± 0.009 a | 0.060 ± 0.012 ab |  |
| Reference soil | 0.09 ± 0.02 a | 0.09 ± 0.02 a | 0.01 ± 0.01 a | 0.066 ± 0.016 b | 0.002 ± 0.001 a | 0.959 ± 0.111 b | 0.068 ± 0.024 a | 0.056 ± 0.007 ab |  |
